# The Impact of Iron Dyshomeostasis and Anaemia on Long-Term Pulmonary Recovery and Persisting Symptom Burden after COVID-19: A Prospective Observational Cohort Study

**DOI:** 10.3390/metabo12060546

**Published:** 2022-06-14

**Authors:** Thomas Sonnweber, Philipp Grubwieser, Sabina Sahanic, Anna Katharina Böhm, Alex Pizzini, Anna Luger, Christoph Schwabl, Sabine Koppelstätter, Katharina Kurz, Bernhard Puchner, Barbara Sperner-Unterweger, Katharina Hüfner, Ewald Wöll, Manfred Nairz, Gerlig Widmann, Ivan Tancevski, Judith Löffler-Ragg, Günter Weiss

**Affiliations:** 1 Department of Internal Medicine II, Medical University of Innsbruck, 6020 Innsbruck, Austria; philipp.grubwieser@student.i-med.ac.at (P.G.); sabina.sahanic@i-med.ac.at (S.S.); anna-k.boehm@i-med.ac.at (A.K.B.); alex.pizzini@tirol-kliniken.at (A.P.); sabine.koppelstaetter@tirol-kliniken.at (S.K.); katharina.kurz@i-med.ac.at (K.K.); bernhard.puchner@tirol-kliniken.at (B.P.); manfred.nairz@gmail.com (M.N.); ivan.tancevski@tirol-kliniken.at (I.T.); judith.loeffler@i-med.ac.at (J.L.-R.); 2 Department of Radiology, Medical University of Innsbruck, 6020 Innsbruck, Austria; anna.luger@i-med.ac.at (A.L.); christoph.schwabl@i-med.ac.at (C.S.); gerlig.widmann@i-med.ac.at (G.W.); 3 Department of Psychiatry, Psychotherapy, Psychosomatics and Medical Psychology, University Clinic for Psychiatry II, 6020 Innsbruck, Austria; barbara.sperner-unterweger@i-med.ac.at (B.S.-U.); katharina.huefner@i-med.ac.at (K.H.); 4 Department of Internal Medicine, St. Vinzenz Hospital, 6511 Zams, Austria; ewald.woell@krankenhaus-zams.at; 5 Christian Doppler Laboratory for Iron Metabolism and Anemia Research, Medical University of Innsbruck, Anichstrasse 35, 6020 Innsbruck, Austria

**Keywords:** COVID-19, SARS-CoV-2, inflammation, iron metabolism, hyperferritinaemia, hepcidin, post-acute sequelae of COVID (PASC), long-COVID

## Abstract

Coronavirus disease 2019 (COVID-19) is frequently associated with iron dyshomeostasis. The latter is related to acute disease severity and COVID-19 convalescence. We herein describe iron dyshomeostasis at COVID-19 follow-up and its association with long-term pulmonary and symptomatic recovery. The prospective, multicentre, observational cohort study “Development of Interstitial Lung Disease (ILD) in Patients With Severe SARS-CoV-2 Infection (CovILD)” encompasses serial extensive clinical, laboratory, functional and imaging evaluations at 60, 100, 180 and 360 days after COVID-19 onset. We included 108 individuals with mild-to-critical acute COVID-19, whereas 75% presented with severe acute disease. At 60 days post-COVID-19 follow-up, hyperferritinaemia (35% of patients), iron deficiency (24% of the cohort) and anaemia (9% of the patients) were frequently found. Anaemia of inflammation (AI) was the predominant feature at early post-acute follow-up, whereas the anaemia phenotype shifted towards iron deficiency anaemia (IDA) and combinations of IDA and AI until the 360 days follow-up. The prevalence of anaemia significantly decreased over time, but iron dyshomeostasis remained a frequent finding throughout the study. Neither iron dyshomeostasis nor anaemia were related to persisting structural lung impairment, but both were associated with impaired stress resilience at long-term COVID-19 follow-up. To conclude, iron dyshomeostasis and anaemia are frequent findings after COVID-19 and may contribute to its long-term symptomatic outcome.

## 1. Introduction

Iron homeostasis is regulated by various inputs, including erythropoiesis, hypoxia and inflammation [1,2,3,4]. In coronavirus disease 2019 (COVID-19), dysregulation of iron homeostasis is frequently found and has been related to the induction of pro-inflammatory signalling pathways such as the interleukin-6 (IL6)/signal transducer and activator of transcription 3 (STAT3) cascade [5,6,7,8,9,10]. IL6 expression is typically high during acute COVID-19, especially in severe disease, and induces hepcidin production. Hepcidin serves as the master regulator of iron homeostasis, as it blocks cellular iron excretion via the degradation of the sole cellular ferrous iron exporter ferroportin-1 (Fpn-1) [11,12,13]. The latter results in decreased nutritional iron uptake in the duodenum, iron sequestration within the monocyte-macrophage system (MPS) and increased production of the main cellular iron storage protein ferritin [14,15]. Whereas this adaption is an essential part of nutritional immunity and may hamper the virulence of severe acute respiratory syndrome coronavirus 2 (SARS-CoV-2), it also has some downsides, as persisting iron deprivation results in impaired haematopoiesis [16,17]. Accordingly, both anaemia of inflammation (AI) and iron deficiency anaemia (IDA) are frequently found in severe COVID-19 and may contribute to the morbidity and mortality of the acute disease [10,18,19,20]. Additionally, local iron accumulation, e.g., in inflamed pulmonary or cardiac tissue, may contribute to COVID-19-related organ damage. An overload of local iron-binding protein capacities facilitates the emergence of free ferrous iron, which catalyses the formation of toxic radicals via the Fenton reaction, thereby contributing to tissue damage [21]. Accordingly, histological evaluations described iron accumulation in COVID-19-induced lung fibrosis and the left ventricular myocardium of patients who died from COVID-19 [22,23,24].

The high prevalence of iron dyshomeostasis in COVID-19 was recognized early on, as hypoferremia and hyperferritinaemia are frequent findings in COVID-19 patients [7,25]. In particular, severe courses of COVID-19 are typically associated with hyperferritinaemia, and iron dyshomeostasis is a risk factor for an unfavourable COVID-19 outcome [26,27,28,29,30,31,32]. To date, it is still not clear if COVID-19-related alterations of iron handling are only a reflection of the host adapting to the acute infection or if SARS-CoV-2 per se interacts with iron homeostasis, and thus iron dyshomeostasis is a pathognomic feature of COVID-19. The latter theory is supported by the observation that hyperferritinaemia can be disproportionally high during COVID-19 and may not be solely explained by inflammatory induction. In this context, it has been recently shown that a spike protein fragment of SARS-CoV-2, which is referred to as “covidin”, may mimic the biological function of hepcidin and alter iron homeostasis independently of inflammatory triggers [22]. In addition, SARS-CoV-2 infects red blood cell progenitor cells, thus the high prevalence of anaemia during acute COVID-19 may not only be related to the systemic inflammatory reaction but also a SARS-CoV-2-related alteration of iron handling and haemoglobin synthesis in the bone marrow [18,33,34]. On the other hand, severe COVID-19 is associated with massive interferon-gamma formation, which induces ferritin formation as also seen in the somehow similar but pathophysiological distinct hemophagocytosis syndrome [35,36,37]. In addition, anti-inflammatory treatment significantly improves iron dyshomeostasis in COVID-19, supporting the theory of a mainly inflammation-driven alteration of iron homeostasis in COVID-19 [18]. Finally, the role of hyperferritinaemia in COVID-19 is still related to many ambiguities. Ferritin is the most relevant cellular iron storage protein and is induced by both cellular iron loading and inflammatory cytokines [14,38]. Whereas the role of intracellular ferritin for iron storage and cellular iron sequestration is well characterized, the physiological role of serum ferritin, and especially of hyperferritinaemia during infection, remains elusive. Whereas some argue that hyperferritinaemia may just be an “innocent bystander”, this theory is challenged by the observation of various immunological functions of the H-ferritin subunit, including immunomodulatory and tissue-protective functions [6,7,29,39,40,41,42]. For instance, ferritin per se exerts pro-inflammatory activity and may contribute to hyperinflammation during COVID-19 [43].

Considering these ambiguities concerning COVID-19-related iron dyshomeostasis we previously investigated the role of iron homeostasis and haematopoiesis during acute COVID-19 and early post-acute COVID-19 follow-up [10,18,20]. In these studies, we reported on the significant prognostic impact of iron deficiency and anaemia in acute COVID-19 and the association of persisting inflammation, iron dyshomeostasis and impaired pulmonary recovery at the early post-acute follow-up after COVID-19.

We herein shed light on the long-term alterations of haematopoiesis and iron metabolism after COVID-19 and their association with the recovery after acute COVID-19. For this, we present long-term follow-up data prospectively assessed in the “Development of Interstitial Lung Disease (ILD) in Patients With Severe SARS-CoV-2 Infection (CovILD)” trial [20,44,45].

## 2. Results

### 2.1. Patient Characteristics

The CovILD study included 145 COVID-19 patients, who have been comprehensively characterized [20,44,45]. The herein-presented analysis only includes patients who attended all follow-up visits at 60, 100, 180 and 360 days after COVID-19 onset (N = 108). This sub-cohort encompassed predominately middle-aged (mean age = 56 years (SD ± 14 yrs)) male patients (58%) with severe acute COVID-19 who had mainly been hospitalized (hospitalization rate: 75%, ICU care: 25% of the participants). The mean duration of hospital stay was 14 days (SD ± 16 days). Detailed characteristics of the cohort are depicted in Table 1.

### 2.2. Iron Deficiency and Anaemia in Patients Recovering from COVID-19

**Figure 1:**
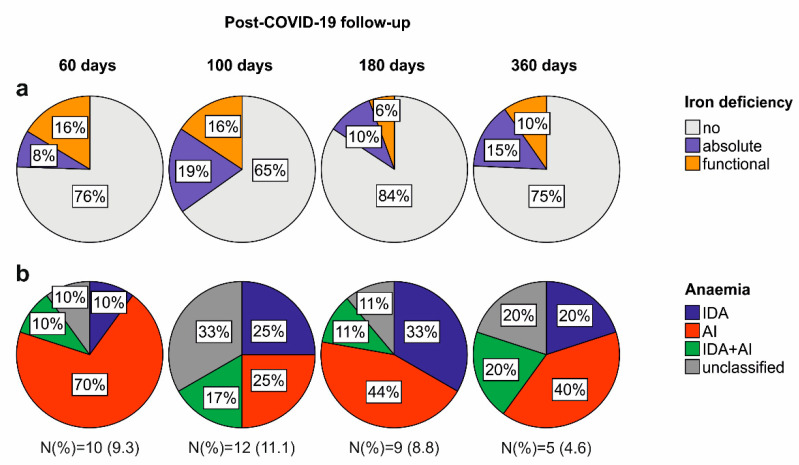
Frequency and phenotype of iron deficiency and anaemia during COVID-19 convalescence. (**a**) Prevalence and phenotype of iron deficiency 60, 100, 180 and 360 days after COVID-19 onset. (**b**) Prevalence and phenotype of anaemia 60, 100, 180 and 360 days after COVID-19 onset. N (%) indicates the total N of anaemic patients and the relative portion of the total cohort for each time point.

At post-acute COVID-19 follow-up, the prevalence of iron deficiency without anaemia, combining absolute and functional iron deficiency, ranged from 35% to 16% of the cohort. Anaemia was found in 11.1% to 4.6% of patients, respectively (Figure 1a). Longitudinally, the prevalence of iron deficiency only slightly dropped (27.8% at 60 days vs. 25.0% at 360 days), whereas the rate of anaemia significantly declined (9.3% at 60 days vs. 4.6% at 360 days). Notably, we found a change in the iron deficiency and anaemia phenotype during the observation period. At early post-COVID-19 follow-up, functional iron deficiency was the predominant finding (N = 20, 18.5%), and only a few individuals suffered from absolute iron deficiency (N = 10, 9.3%), whereas at long-term follow-up, absolute iron deficiency became the prominent iron deficiency phenotype (absolute iron deficiency: N = 17, 15.7%, functional iron deficiency: N = 10, 9.3%). In line with the iron deficiency phenotype, AI was the most dominant form of anaemia, especially at early post-acute follow-up, whereas IDA, or combined IDA and AI, or unclassifiable/multifactorial anaemia were more prevalent at the 360 days follow-up (Figure 1b). Of note, anaemia was predominantly mild, as reflected by the mean haemoglobin levels of anaemic patients of 113 g/L (SD ± 9.4 g/L) at the 60 days post COVID-19 follow-up, and haemoglobin levels gradually increased over the study period (Table 2). Patterns of serum iron markers were consistent with functional iron deficiency at early post-acute COVID-19 follow-up with a translation to absolute iron deficiency at long-term follow-up, as both serum ferritin and hepcidin concentrations were significantly higher 60 days after acute COVID-19 as compared to the one-year re-evaluation (Table 2).

### 2.3. Persisting Inflammation and Hyperferritinaemia at Post-COVID-19 Follow-Up

**Figure 2:**
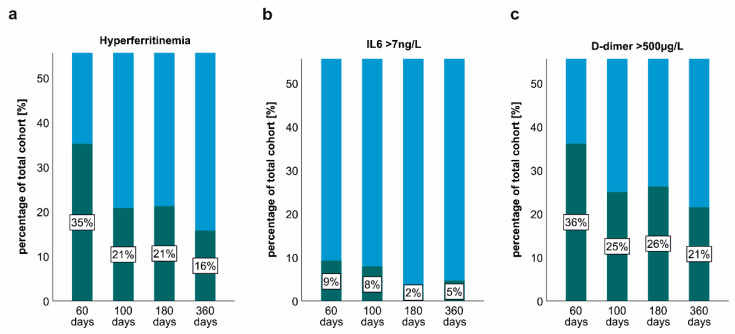
Persisting thrombo-inflammation at post-COVID-19 follow-up. (**a**) Prevalence of hyperferritinaemia 60, 100, 180 and 360 days after COVID-19 onset. (**b**) Persisting elevations of IL6 60, 100, 180 and 360 days after COVID-19 onset. (**c**) Persisting elevations of d-dimer 60, 100, 180 and 360 days after COVID-19 onset. The green colour indicates the relative percentages of the total cohort with hyperferritinaemia, IL6 or D-dimer elevations, whereas the blue colour indicates the relative portion of patients without hyperferritinaemia, IL6 or D-dimer elevations.

At early post-acute COVID-19 follow-up, a substantial portion of study participants presented with low-grade thrombo-inflammation, as reflected by increased IL6, neopterin, pro-calcitonin, C-reactive protein (CRP) or d-dimer blood concentrations (Table 3). At long-term follow-up, inflammatory parameters significantly dropped and only a small subgroup demonstrated signs of persisting inflammation (Table 3). Accordingly, hyperferritinaemia, increased IL6 concentrations and elevations of d-dimer were frequently found at the 60 days post-COVID follow-up and significantly decreased over time (Figure 2). Despite this longitudinal decline, a substantial portion of the patients presented with persisting hyperferritinaemia (N = 17, 15.7%) and d-dimer elevations at the 360-day follow-up (N = 23, 21.3%).

### 2.4. Gender-Related Differences in the Prevalence of Iron Deficiency and Anaemia at Post-COVID-19 Follow-Up

**Figure 3:**
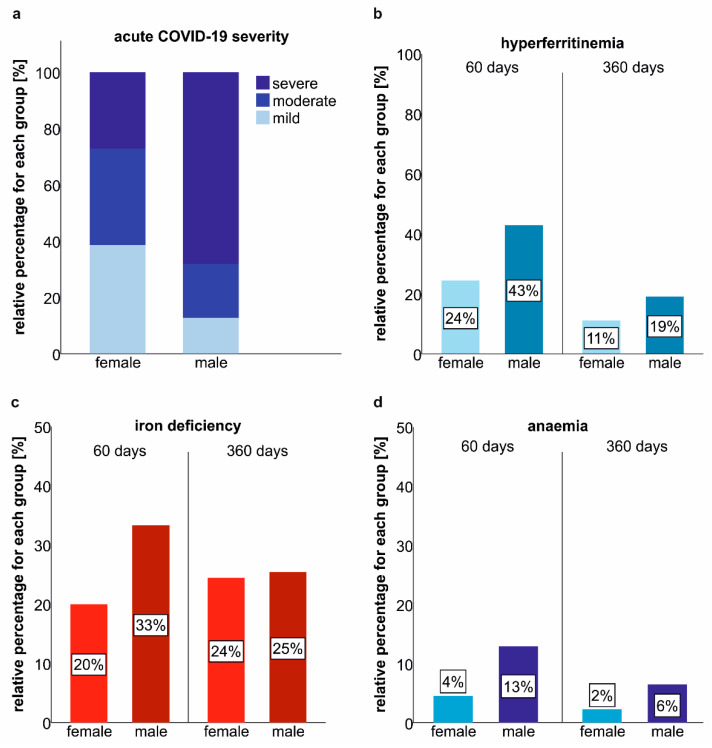
Gender-related differences in iron deficiency and anaemia prevalence after COVID-19. (**a**) Severity of acute COVID-19 according to gender. (**b**) Frequency of hyperferritinaemia according to gender 60 and 360 days after COVID-19 onset. (**c**) Iron deficiency in females and males 60 and 360 days after COVID-19 onset. (**d**) Anaemia in females and males 60 and 360 days after COVID-19 onset.

Male patients suffered from more severe acute COVID-19 as compared to female study participants (Figure 3a). Accordingly, the frequencies of hyperferritinaemia, iron deficiency and anaemia were significantly higher in males as compared to females at early post-COVID-19 follow-up (Figure 3b–d). Until the last follow-up, these gender-specific differences extenuated, but males still tended to have a higher prevalence of iron dyshomeostasis and anaemia.

### 2.5. Association of Persisting Inflammation, Iron Dyshomeostasis and Anaemia

Disturbances of iron homeostasis, including hyperferritinaemia and the emergence of anaemia, during acute COVID-19 have been mainly attributed to inflammation. Thus, we evaluated associations between persisting inflammation and iron dyshomeostasis. Still, at least at the systemic level disturbances of iron homeostasis including ferritin elevations were only weakly to moderately associated with thrombo-inflammatory parameters such as IL6, CRP, procalcitonin, neopterin or d-dimer concentrations (Table 4). At follow-up, both iron dyshomeostasis and systemic inflammation were less frequent, and inflammatory parameters were only moderately associated with serum iron and TSAT. Interestingly, when comparing individuals with and without iron deficiency, iron-deficient patients demonstrated significantly higher inflammatory serum parameters and hepcidin concentrations over the study period. For instance, at the 360 days follow-up, patients with iron deficiency displayed higher CRP (*p* = 0.024), neopterin (*p* = 0.001) and hepcidin (*p* = 0.006) concentrations in the blood as compared to individuals without iron deficiency, whereas patients with absolute iron deficiency tended to have the highest concentrations of these inflammation markers.

### 2.6. Association of Iron Dyshomeostasis and Anaemia with Clinical COVID-19 Severity and Structural Lung Recovery

**Figure 4:**
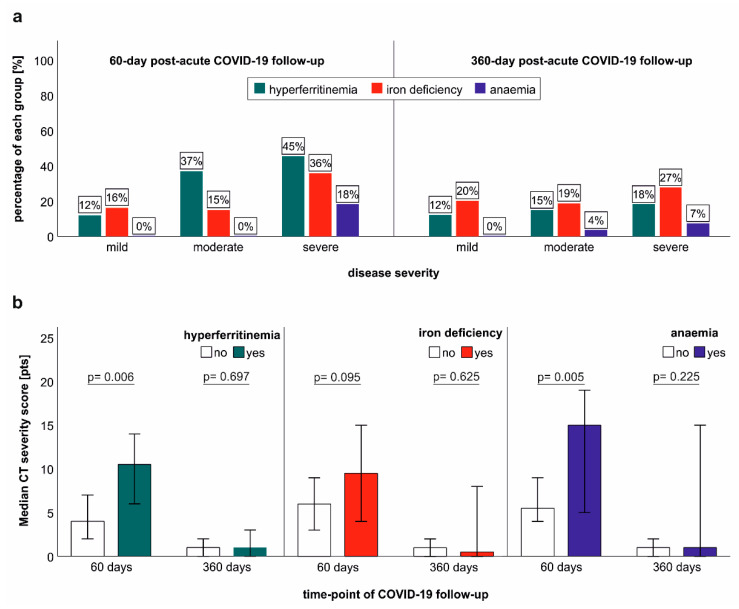
Association of acute COVID-19 severity with iron dyshomeostasis and anaemia at early and long-term COVID-19 follow-up. (**a**) Frequency of hyperferritinaemia, iron deficiency and anaemia at 60 days and 360 days post-COVID-19 follow-up. (**b**) Structural lung abnormalities were assessed with computed tomography (CT) in patients with hyperferritinaemia, iron deficiency and anaemia at early and long-term post-COVID-19 follow-up. Error bars indicate 95% confidence intervals; *p*-values are depicted according to the Mann-Whitney-U test.

Iron dyshomeostasis and anaemia were significantly related to the severity of acute COVID-19 at the early post-acute follow-up, as patients with severe COVID-19 showed a disproportionally high prevalence of hyperferritinaemia, iron deficiency and anaemia. Still, at long-term follow-up, this association considerably weakened (Figure 4a). The severity of structural lung abnormalities was significantly higher in patients with iron dyshomeostasis and anaemia at early post-acute follow-up (Figure 4b). Although individuals with iron dyshomeostasis and anaemia demonstrated a higher variability of structural lung involvement at long-term post-COVID-19 follow-up, we did not find a significant difference in the severity of structural lung impairment between patients with or without iron dyshomeostasis or in association with anaemia at long term follow-up (Figure 4b). Conclusively, whereas hyperferritinaemia, iron deficiency and anaemia were related to acute COVID-19 severity at early post-acute follow-up; this relation was not apparent at long-term follow-up.

### 2.7. Impact of Iron Dyshomeostasis and Anaemia on Long-Term Symptom Burden and Exercise Tolerance

**Figure 5:**
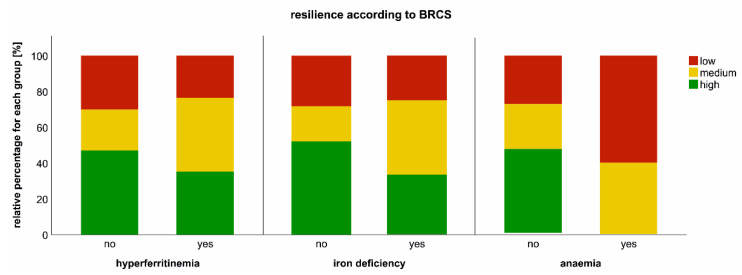
Impact of iron dyshomeostasis and anaemia on resilience at long-term post-COVID-19 follow-up. Resilience was evaluated with Brief Resilient Coping Scale (BRCS, low (4–13 pts), medium (14–16 pts) and high resilience (17–20 pts)) at the 360 days post-COVID reassessment according to the presence of hyperferritinaemia, iron deficiency or anaemia are depicted.

Iron dyshomeostasis and anaemia are not only relevant findings during acute COVID-19 but may also be of importance for post-COVID-19 recovery [20]. Thus, we investigated the association of iron dysbalance and anaemia with patients’ resilience coping, fatigue, quality of life (QoL) and exercise capacity at long-term follow-up. One year after COVID-19 onset, a relevant sub-cohort of patients demonstrated chronic impairments as far as persistence of fatigue, impairment of the QoL and exercise capacity are concerned. In detail, the mean European Quality of Life visual analogue scale (EQ-VAS), which provides patients’ QoL self-reports, was 85% (CI 79.7–85.0%), whereas 100% would describe a perfect QoL. The Chalder Fatigue Scale (CFS) assessment, which reflects the severity of tiredness in patients with fatigue, resulted in a median Likert score of 12 points (CI 12.4–15.0 points), whereas 37% exceeded a score of 14—indicating increased fatigue— and two patients demonstrated a CFS score equal or above 28, which is indicative for chronic fatigue syndrome. Accordingly, 8.8% (N = 9) of patients displayed a reduced six-minute walking distance. Notably, resilience was impaired in a substantial portion of the CovILD cohort, as only 45% had high resilience, whereas 26% and 29% demonstrated medium and low resilience, respectively. Interestingly, whereas fatigue, QoL and exercise capacity were not significantly related to iron dyshomeostasis or anaemia, a reduction in resilience was associated with persisting hyperferritinaemia, iron deficiency and anaemia at long-term follow-up (Figure 5).

## 3. Discussion

A disequilibrium of iron homeostasis is a significant laboratory finding of COVID-19. Most prominently, hyperferritinaemia and hypoferremia are frequently observed during acute COVID-19 and have been described as prognostic markers for COVID-19 outcome [7,9,25,26,27,28,30,31,46,47]. Accordingly, we previously reported an association between the resolution of structural pulmonary impairment and the persistence of hyperferritinaemia and inflammation at early post-acute COVID-19 follow-up [20,44]. Herein, we shed light on the long-term recovery after COVID-19 focusing on iron dyshomeostasis and anaemia post-COVID-19. Of interest, whereas anaemia was a frequent finding at early post-acute follow-up, we discovered a significant change in the anaemia phenotype and frequency at follow-up. Most notably, anaemia at the 60 days follow-up mainly demonstrated features of AI, suggesting that COVID-19-related inflammation followed by hepcidin-induced iron sequestration and functional iron deficiency is the major trigger for anaemia during acute and early post-acute COVID-19. This theory is supported by the herein-presented observation of a high frequency of hyperferritinaemia and increased hepcidin concentrations at the 60 days post-COVID-19 follow-up as well as previously published data describing a high prevalence of AI in COVID-19 patients [10,18,48]. At long-term follow-up, the prevalence of anaemia significantly decreased. This observation is explained by the resolution of inflammation during COVID-19 convalescence, as reflected by decreasing inflammatory markers such as IL6, procalcitonin and CRP, as well as a restoration of iron homeostasis, mirrored by a drop of hepcidin and ferritin levels. Still, a substantial portion of patients demonstrated long-term impairment of iron handling resulting in the emergence of IDA and combined forms of anaemia (IDA + AI). Although this observation urges further mechanistic evaluation, a prolonged disturbance of iron absorption following COVID-19 might be a possible explanation for the development of absolute iron deficiency as well as IDA at long-term follow-up.

The observation of differential phenotypes of anaemia at post-COVID-19 follow-up warrants a thorough characterization of the iron status, as treatment of anaemia depends on the precise assessment of the anaemia phenotype [49]. For instance, IDA without significant signs of persisting inflammation may be successfully treated with iron supplementation, whereas iron substitution is less effective for patients with AI and systemic inflammation at COVID-19 follow-up [17].

Interestingly, the prevalence of ID and anaemia was higher in males as compared to females recovering from COVID-19. This is in contrast to the prevalence of iron deficiency and anaemia in the general population, where both conditions are far more frequent in females, suggesting that iron dyshomeostasis and anaemia are related to COVID-19 and disease severity [50,51]. Accordingly, iron deficiency and anaemia at follow-up were related to acute COVID-19 severity, and males tended to have higher markers of systemic inflammation during follow-up (data not shown).

Both iron deficiency and anaemia are well-known contributors to patients’ morbidity [17,52,53]. For instance, exercise capacity is impaired in individuals with iron deficiency, even without the development of anaemia, which may be partly linked to reduced mitochondrial functionality [54,55,56,57]. However, inflammation and iron loading can trigger radical formation, thereby resulting in mitochondrial dysfunction as well [58,59]. Nonetheless, iron deficiency impacts mental health, fatigue and quality of life [60]. Fatigue and reduced exercise capacity are frequently observed in patients recovering from COVID-19 and thus are often described in individuals diagnosed with post-acute sequelae of COVID (PASC) or long-COVID [47,61]. Notably, these diagnostic terms still suffer from blurry definitions and uncertain clinical value. Objective measures often fail to describe somatic causes of the symptom burden of these individuals and the overall understanding of the multifactorial causes of PASC is still rudimentary [47]. Given the high frequency of iron dyshomeostasis during acute COVID-19 and at post-acute COVID-19 follow-up, we hypothesized that alterations in iron metabolism and haematopoiesis might contribute to the persisting symptom burden post-COVID-19. As fatigue and impaired exercise capacity are among the most frequent persisting symptoms at COVID-19 follow-up, we focused on these outcomes in the CovILD cohort and assessed the impact of iron dyshomeostasis and anaemia on these outcomes [62]. PASC are reported at a high prevalence and are related to acute COVID-19 severity [47,61]. The CovILD trial mainly included severe COVID-19 patients; thus, we expected a high rate of PASC connected with a significant impairment of QoL, exercise capacity and a substantial rate of chronic fatigue [45]. Still, at the 360 days post-COVID-19 follow-up, the observed impairment of QoL and exercise capacity was relatively mild, and high CFS scores were only found in a few individuals. Although fatigue and exercise impairment are considered to be major symptoms of PASC, our herein-presented evaluation does not reflect a severe impairment at long-term post-COVID-19 follow-up. This finding is striking and may be explained by two considerations. First, the interpretation of the clinical significance of PASC is still related to various ambiguities, as the definition of PASC includes a variety of different symptoms and typically does not describe the clinical significance of the assessed symptoms. Secondly, the prevalence of PASC may decrease over time, and PASC may be reversible in most patients.

Iron deficiency was frequently found in the study participants and persisted in 25% of the patients until the 360 days post-COVID-19 follow-up. Additionally, 5% of the study participants suffered from anaemia at the long-term follow-up. Interestingly, neither iron deficiency nor anaemia significantly impaired exercise capacity. The latter may be explained by the fact that hardly any patient developed severe anaemia or iron deficiency, and the predominantly mild anaemia and iron deficiency may not significantly impact exercise capacity in a low-intensity exercise test, such as the herein applied six-minute walking test. Accordingly, QoL and fatigue were not significantly influenced by mild anaemia. Still, when we evaluated patients’ stress coping capacities, iron deficiency and even more pronounced anaemia were associated with reduced resilience. This finding is of high interest, as it points toward an impact of iron deficiency and anaemia on convalescence in post-COVID-19 patients who frequently report neuropsychiatric symptoms, including sleeping disorders and impairment of neurocognition [63,64]. Accordingly, iron deficiency and anaemia are risk factors for developing a psychiatric disorder, and psychiatric disorders are generally associated with lower levels of resilience compared to mentally healthy controls [65,66]. Mechanistically, it has been suggested that iron deficiency induces poor myelination or disruptions in neurotransmitter levels or mitochondrial activity [67,68].

Finally, we must acknowledge that the herein-presented study has some limitations. First, the observational study design reports associations rather than causality, and mechanistic studies are needed to provide further insight into involved signalling pathways and a potential cause-effect relationship. Second, the prevalence of iron deficiency and anaemia in the general population is high, and according to the study design, we cannot assess the prevalence of anaemia or iron deficiency in the CovILD cohort before COVID-19 onset. Third, treatment effects such as iron supplementation were not investigated in this study. Fourth, the role of chronic low-grade inflammation and its interaction with iron metabolism and haematopoiesis cannot be fully evaluated with the presented study design, as low-grade inflammation may not be assessed by the analysis of inflammatory biomarkers at the systemic level. Thus, at this point, we cannot rule out or confirm if persisting iron dyshomeostasis is functionally related to chronic low-grade inflammation at long-term follow-up.

## 4. Materials and Methods

### 4.1. Patients and Study Design

The CovILD study is a prospective multi-centre observational cohort trial which included 145 COVID-19 patients (ClinicalTrials.gov number, NCT04416100). The study longitudinally assessed post-COVID-19 recovery 60, 100, 180 and 360 days after disease onset. Medical history, acute disease severity and symptom burden were assessed, and each visit included a clinical examination, a standardized questionnaire evaluating COVID-19-related symptoms, performance testing (e.g., six-minute walking test (SMWT)), structural lung evaluation using CT without contrast agent, lung function testing and the acquisition of blood for laboratory analyses. Inclusion criteria were an age of 18 years or older, COVID-19 diagnosis established by typical clinical symptoms for COVID-19, a positive reverse transcription-polymerase chain reaction (RT-PCR) SARS-CoV-2 result obtained from a nasopharyngeal or oropharyngeal swab and the ability to perform repetitive follow-up visits at the primary study centre at the Medical University of Innsbruck. Participants were recruited at three participating study centres, namely the St. Vinzenz hospital in Zams, the post-COVID rehabilitation Center of Münster and the Medical University of Innsbruck. Patients with mild (out-patient treatment), moderate (in-hospital treatment without oxygen supply or ventilation), and severe (in-hospital treatment with the need for oxygen supplementation or intensive care unit (ICU) treatment) acute COVID-19 were included. Informed written consent was obtained from all participants, and the study was approved by the local ethics committee at the Medical University of Innsbruck (EK Nr: 1103/2020).

### 4.2. Laboratory Assessment

Blood samples were collected at each follow-up and obtained via routine peripheral vein puncture. Laboratory evaluation was performed with standardized ISO-certified procedures as previously described [44,45]. Blood gas analysis was performed via punctuation of the hyperperfused earlobe following Finalgon application.

### 4.3. Diagnosis of Anaemia, Iron Deficiency and Hyperferritinaemia

Iron studies were performed at each follow-up and included serum iron, TSAT, serum ferritin, sTFR and the calculation of the sTFRF index. Iron deficiency was defined by TSAT and serum ferritin and further characterized as absolute iron deficiency (TSAT < 20%, serum ferritin < 100 µg/L) or functional iron deficiency (TSAT < 20%, serum ferritin > 100 µg/L) [69,70,71,72]. Hyperferritinaemia was defined by serum ferritin >200 µg/L for women and >300 µg/L for men, as previously reported [73].

Females were diagnosed with anaemia if haemoglobin (Hb) concentrations were below 120 g/L, whereas males were considered anaemic if Hb was below 130 g/L. In addition, anaemia was characterized as IDA (sTFRF index > 2, TSAT < 20%, serum ferritin < 30 µg/L)), AI (TSAT < 20% and serum ferritin > 100 µg/L or serum ferritin 30–100 µg/L and sTFRF index < 1), combined IDA and AI (IDA + AI TSAT < 20%, serum ferritin 30–100 µg/L, sTFRF index > 2) or unclassifiable anaemia (TSAT normal or reduced, serum ferritin > 30 µg/L, sTFRF index 1–2), as previously described [20,74,75].

### 4.4. Structural Lung Evaluation with CT

A low-dose (100 kVp tube potential) CT scan of the chest without the use of an iodine contrast agent was performed at each follow-up. We used a 128-slice multidetector CT hardware with a 38.4 × 0.6 mm collimation and spiral pitch factor of 1.1 (SOMATOM Definition Flash, Siemens Healthineers, Erlangen, Germany).

Structural lung evaluation was performed by three radiologists experienced in lung CT diagnostics. The analysis included pattern description (ground-glass opacities (GGO), consolidations, bronchiectasis and reticulations as defined by the glossary of terms of the Fleischner Society) and severity grading [76]. The severity grading was independently analysed by three radiologists. The latter used the following standardized scoring system: Each lung lobe was graded according to the presence of pulmonary abnormalities: 0—none; 1—minimal (subtle GGO, very few findings); 2—low (several GGO, subtle reticulation); 3—moderate (multiple GGO, reticulation, small consolidation); 4—marked (extensive GGO, consolidation, reticulation with distortion) and 5—massive (massive findings, parenchymal destructions), and a total lung score was obtained via the addition of the scores for each lobe, resulting in a score ranging from 0 to 25 points (i.e., a maximum score of 25 if each lobe was scored 5 points).

### 4.5. Assessment of Exercise Capacity, Resilience, Fatigue and Quality of Life

Exercise capacity was evaluated with the SMWT. Additionally, we used standardized questionnaires to assess resilient coping (BRCS), fatigue (CFS) and quality of life (European Quality of Life 5 Dimensions 5 Level Version and the EQ-VAS) [77,78,79].

### 4.6. Statistical Analysis

Statistical analyses were performed with IBM SPSS Statistics version 27.0.1.0 (IBM, Chicago, IL, USA). Descriptive data analysis included tests for homoscedasticity and data distribution (Levene test, Kolmogorov–Smirnov test, Shapiro–Wilk test and density blot/histogram analysis). Group comparisons of continuous data were assessed with the Mann–Whitney-U test and Kruskal–Wallis test for continuous data and Fisher’s exact test or Chi-Square test for binary and categorical data. Multiple testing was adjusted by the Sidak formula, as appropriate. Correlation analysis was performed with the Spearman rank test.

## 5. Conclusions

Alterations of iron homeostasis and anaemia are frequent findings in acute COVID-19 but also at post-acute COVID-19 follow-up. During COVID-19 convalescence, systemic thrombo-inflammation, hyperferritinaemia and the prevalence of anaemia gradually decline; still, a significant subgroup of the post-COVID population displays persisting iron deficiency or even anaemia, which may contribute to persisting symptom burden in these individuals. A link between iron dyshomeostasis and SARS-CoV-2 driven inflammation is evident and may at least partly contribute to this finding, but the interconnection of COVID-19 pathobiology and iron dyshomeostasis is likely far more complex and urges further evaluation.

## Figures and Tables

**Table 1:** Characteristics of the study cohort (N = 108).

**Demographics**	
Mean age–years (SD)	56 (14)
Female sex–no. (%)	45 (42)
Median body mass index (SD) ^1^	26.4 (4.5)
Smoking history–no. (%)	39 (36)
**Comorbidities–No. (%)**	
None	27 (25)
Cardiovascular disease	42 (39)
Hypertension	29 (27)
Pulmonary disease	19 (18)
Endocrine disease	63 (58)
Diabetes mellitus, type 2	17 (16)
Chronic kidney disease	7 (6)
Chronic liver disease	6 (6)
Malignancy	10 (9)
Immunodeficiency ^2^	5 (5)
**Treatment during acute COVID-19 ^3^**	
Hospitalization–no. (%)	81 (75)
Oxygen supply–no. (%)	54 (50)
Non-invasive ventilation–no. (%)	2 (2)
Invasive ventilation–no. (%)	25 (23)

^1^ The body mass index is the weight (kilograms) divided by the square of the height in meters; ^2^ due to pre-existing disease or ongoing immunosuppressive treatment; ^3^ all patients needing non-invasive or invasive ventilation were supplied with oxygen before ICU admission.

**Table 2:** Iron studies and haemogram during COVID-19 convalescence.

Time after COVID-19 Onset	60 Days	100 Days	180 Days	360 Days	*p*-Value	Effect Size
serum iron–µmol/L (SD)	16.0 (6.0)	15.2 (5.3)	17.7 (5.6)	17.3 (5.9)	0.055	0.187
TSAT–% (SD) ^1^	26 (11)	24 (10)	27 (9)	26 (9)	0.804	0.240
serum ferritin–µmol/L (SD)	269 (251)	198 (197)	183 (153)	198 (191)	<0.001	−0.443
hepcidin-25–µg/L (SD)	18.9 (13.8)	15.7 (13.4)	18.2 (14.1)	13.0 (10.2)	<0.001	−0.526
sTFR–mg/L (SD) ^2^	3.4 (1.1)	3.2 (1.0)	2.9 (1.1)	3.0 (0.8)	<0.001	−0.477
ferritin index–value (SD) ^3^	1.6 (0.7)	1.7 (1.0)	1.5 (1.3)	1.5 (0.6)	0.100	−0.161
haemoglobin–g/L (SD)	139 (14)	141 (16)	144 (15)	146 (14)	<0.001	−0.783
leucocytes–G/L (SD)	6.44 (2.73)	6.28 (2.07)	6.13 (1.65)	6.07 (1.61)	0.011	−0.250
thrombocytes–G/L (SD)	264 (74)	247 (64)	240 (61)	243 (54)	<0.001	−0.338

^1^ transferrin saturation; ^2^ soluble transferrin receptor; ^3^ soluble transferrin receptor/log serum ferritin; p-value depicts differences between 60 days and 360 days follow-up as calculated by paired *t*-test; effect size according to Cohen’s d. N = 108.

**Table 3:** Time course of serum markers of thrombo-inflammation in the CovILD cohort.

Time after COVID-19 Onset	60 Days	100 Days	180 Days	360 Days	*p*-Value	Effect Size
CRP–mg/dL (SD) ^1^	0.37 (1.12)	0.29 (0.68)	0.21 (0.50)	0.36 (0.90)	0.847	−0.019
Procalcitonin–µg/L (SD)	0.07 (0.02)	0.07 (0.03)	0.02 (0.04)	0.02 (0.06)	<0.001	−1.376
IL6–ng/L (SD)	3.4 (5.5)	3.2 (2.6)	1.9 (2.3)	1.3 (2.7)	<0.001	−0.410
Neopterin–nmol/L (SD)	9.7 (4.5)	8.4 (2.9)	9.0 (3.7)	10.2 (6.7)	0.360	0.089
D-dimer–µg/L (SD)	575 (541)	572 (891)	470 (513)	363 (202)	<0.001	−0.443

^1^ C-reactive protein; *p*-value depicts differences between 60 days and 360 days follow-up as calculated by paired *t*-test; effect size is depicted according to Cohen’s d calculation, N = 108.

**Table 4:** Correlation of serum markers of thrombo-inflammation and iron parameters at early post-acute and long-term follow-up.

Serum Marker	Iron	TSAT ^3^	Ferritin	sTFR ^4^	sTFRF Index ^5^	Hepcidin
	**60 days post-COVID-19 follow-up**					
CRP–ρ ^1^	−0.198 *	−0.272 **	0.040	0.175	0.095	−0.006
Procalcitonin–ρ	−0.085	−0.069	0.089	0.185	0.060	−0.012
IL6–ρ ^2^	−0.202 *	−0.161	0.067	0.272 **	0.213 *	−0.065
Neopterin–ρ	−0.185	−0.158	0.235 *	0.242 *	0.064	0.035
D-dimer–ρ	−0.248 *	−0.242 *	0.028	0.177	0.131	−0.191
	**360 days post−COVID−19 follow−up**					
CRP–ρ ^1^	−0.335 **	−0.334 **	0.055	0.092	0.049	0.035
Procalcitonin–ρ	−0.196 *	−0.141	0.213 *	0.192 *	0.023	0.156
IL6–ρ ^2^	−0.329 **	−0.321 **	0.001	0.030	0.052	0.056
Neopterin–ρ	−0.235 *	−0.178	0.140	0.238 *	0.097	0.111
D-dimer–ρ	−0.121	−0.071	0.006	−0.049	−0.034	0.021

^1^ C-reactive protein; ^2^ interleukin-6; ^3^ transferrin saturation, ^4^ soluble transferrin receptor, ^5^ soluble transferrin receptor/log serum ferritin; N = 108, ρ = correlation coefficient rho according to Spearman rho calculation, * *p* < 0.05, ** *p* < 0.01.

## Data Availability

The data presented in this study are available in the article.
